# Morganella morganii, an Emerging Cause of Bloodstream Infections

**DOI:** 10.1128/spectrum.00569-22

**Published:** 2022-04-25

**Authors:** Kevin B. Laupland, David L. Paterson, Felicity Edwards, Adam G. Stewart, Patrick N. A. Harris

**Affiliations:** a Department of Intensive Care Services, Royal Brisbane and Women’s Hospital, Brisbane, Queensland, Australia; b Queensland University of Technology, Brisbane, Queensland, Australia; c University of Queensland, Faculty of Medicine, University of Queensland Centre for Clinical Research, Brisbane, Queensland, Australia; d Infectious Diseases Unit, Royal Brisbane and Women's Hospital, Brisbane, Queensland, Australia; e Department of Microbiology, Pathology Queensland, Brisbane, Queensland, Australia; Louis Stokes Cleveland VAMC

**Keywords:** bloodstream infection, epidemiology, *Morganella morganii*

## Abstract

Although recent reports of extensively antibiotic-resistant strains have highlighted the importance of Morganella morganii as an emerging pathogen, the epidemiology of serious infections due to this organism is not well defined. The objective of this study was to determine the incidence, determinants, and outcomes of Morganella morganii bloodstream infections (BSIs). Retrospective, population-based surveillance for Morganella morganii BSIs was conducted in Queensland, Australia, in 2000 to 2019; 709 cases were identified, for an annual incidence of 9.2 cases per million population. Most cases were of community onset, with 280 (39.5%) community-associated cases and 226 (31.9%) health care-associated cases. Morganella morganii BSIs were rare in children and young adults, and the incidence increased markedly with advancing age. The most common foci of infection were skin and soft tissue (131 cases [18.5%]), genitourinary (97 cases [13.7%]), and intraabdominal (90 cases [12.7%]). Most patients (580 cases [81.8%]) had at least one comorbid medical illness, with diabetes mellitus (250 cases [35.3%]), renal disease (208 cases [29.3%]), and congestive heart failure (167 cases [23.6%]) being most prevalent. Resistance to one or more of quinolones, co-trimoxazole, aminoglycosides, or carbapenems was observed in 67 cases (9.5%), and this did not change significantly over the study. The 30-day all-cause case fatality rate was 21.2%, and increasing age, nonfocal infection, heart failure, dementia, and cancer were independently associated with increased risk of death. Morganella morganii BSIs are increasing in our population, and elderly male subjects and individuals with comorbidities are at highest risk. Although antibiotic resistance is not a major contributor to the current burden in Queensland, ongoing surveillance is warranted.

**IMPORTANCE** Recent reports of extensively antibiotic-resistant strains have highlighted the importance of Morganella morganii as an emerging pathogen. Despite its present and evolving importance as an agent of human disease, there is a limited body of literature detailing the epidemiology of serious infections due to Morganella morganii. Therefore, the objectives of this study were to examine the incidence and determinants of Morganella morganii BSIs and to examine risk factors for death in a large Australian population in 2000 to 2019.

## INTRODUCTION

Morganella morganii with Proteus and *Providencia* species comprise the tribe *Proteeae* and are occasional causes of bloodstream infections (BSIs) (1). Morganella morganii has chromosomally encoded *bla*_AmpC_, which confers resistance to cephalosporins and penicillins (2). As a result, treatment with quinolones, aminoglycosides, co-trimoxazole, or carbapenems is usually recommended (3–6). However, the potential for Morganella morganii to acquire other resistance determinants has been increasingly recognized (7). Recent studies have found extensively resistant strains in sewage samples and among a range of animal sources (8–13). As a result of its ability to cause invasive disease, the presence of *bla*_AmpC_ and virulence factors, and its propensity to acquire resistance determinants, Morganella morganii has been labeled an emerging “superbug” (6).

Despite its present and evolving importance as an agent of human disease, there is a limited body of literature detailing the epidemiology of serious infections due to Morganella morganii. Although there have been several reported case series of patients with BSIs, these have been limited by small sample size and reporting from single hospitals that are at risk of selection biases (14–18). The objectives of this study were to examine the incidence and determinants of Morganella morganii BSIs and to examine risk factors for death in a large Australian population.

## RESULTS

A total of 709 incident Morganella morganii BSIs were identified among 705 Queensland residents. Four patients had second incident episodes. The majority of the BSIs were of community onset, with 280 (39.5%) community-associated BSIs and 226 (31.9%) health care-associated BSIs. Of the 203 (28.6%) hospital-onset cases, the median time from admission to diagnosis was 8 days (interquartile range [IQR], 5 to 17 days).

### Demographic determinants.

**FIG 1:**
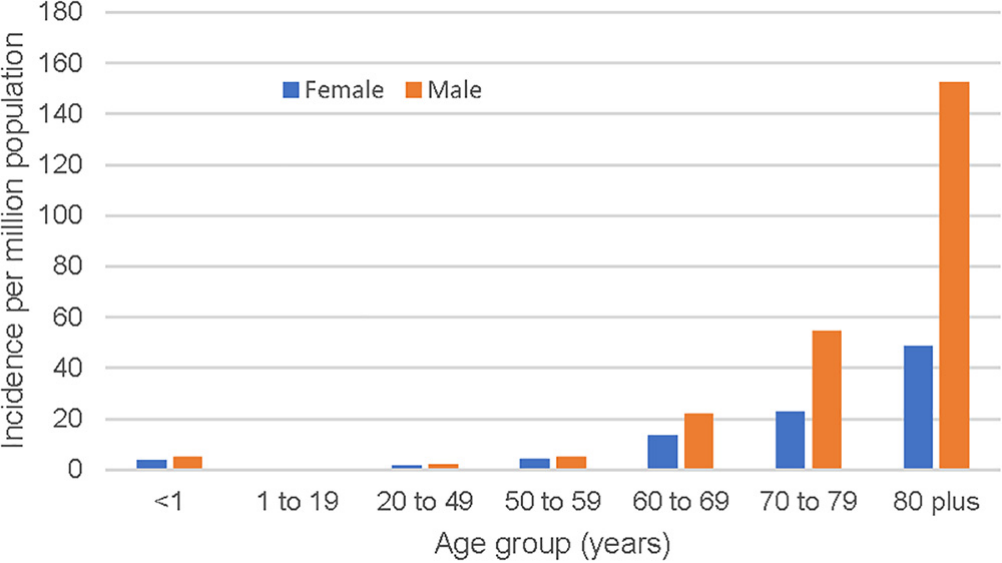
Incidence of Morganella morganii BSIs by age and sex in Queensland, Australia, in 2000 to 2019.

The median age was 75.2 years (IQR, 64.2 to 83.0 years), and two-thirds (463 [65.3%]) of the episodes occurred among male patients. Morganella morganii BSIs were rare in children and young adults, and the incidence increased significantly with older age and male sex, as shown in Fig. 1. Overall, males were at twice the risk for development of Morganella morganii BSIs (10.8 versus 5.7 cases per million; incidence rate ratio [IRR], 1.89 [95% confidence interval [CI], 1.62 to 2.22]; *P* < 0.0001). However, this excess risk among males was due to those 50 years of age and older (32.5 versus 15.8 cases per million; IRR, 2.04 [95% CI, 1.73 to 2.42]; *P* < 0.0001), with no significant excess risk observed among younger individuals (1.40 versus 1.01 cases per million; IRR, 1.38 [95% CI, 0.84 to 2.29]; *P* = 0.18).

### Incidence.

**FIG 2:**
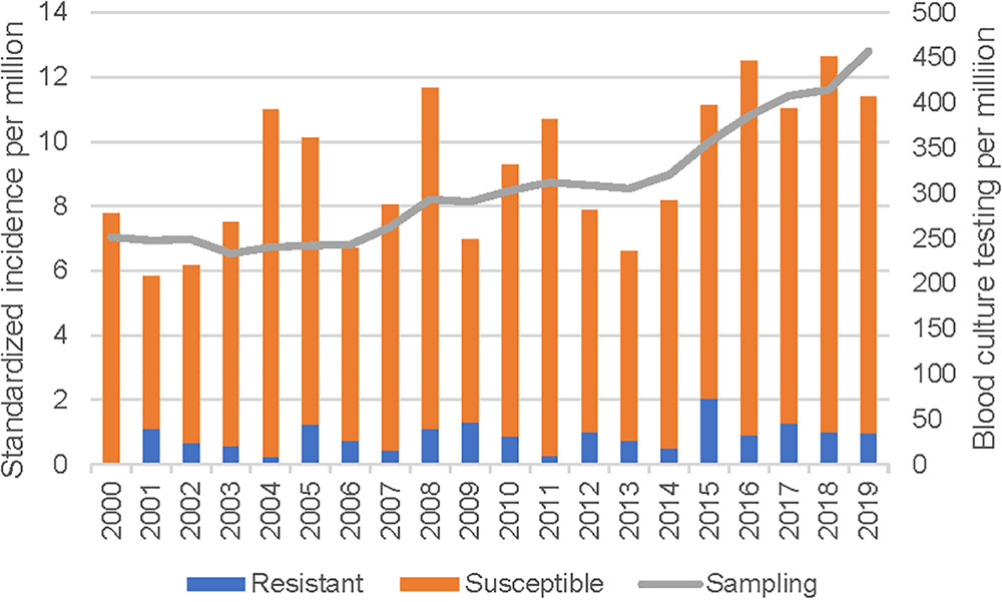
Age- and sex-standardized incidence of Morganella morganii BSIs in Queensland, Australia, in 2000 to 2019.

The overall age- and sex-standardized incidence was 9.2 cases per million annually, with 8.3 and 0.9 cases per million annually for antimicrobial-susceptible and -resistant isolates, respectively. Moderate year-to-year variability was observed in the incidence of Morganella morganii BSIs, as shown in Fig. 2. There was an overall standardized incidence increase of 0.23 cases per million per year, which was associated with an increased blood culture sampling rate during the study (Fig. 2). No significant trend in the incidence of resistant isolates was observed.

### Clinical features.

The clinical characteristics of Morganella morganii BSIs varied considerably by onset category, as shown in Table 1. There was a progressive decrease in age associated with an increasing degree of health care exposure. Community-associated cases had the greatest proportion of males and the lowest median comorbidity scores. The proportions of cases with no comorbid disease varied by onset category and were 35 cases (17.2%), 19 cases (8.4%), and 75 cases (26.8%) for hospital-onset, health care-associated, and community-associated cases, respectively (*P* < 0.001 overall and *P* < 0.02 for each pairwise comparison).

**TABLE 1:** Clinical characteristics of Morganella morganii BSIs according to onset classification

Factor	Data for:				*P*
	Hospital-onset cases (*n* = 203)	Health care-associated cases (*n* = 226)	Community-associated cases (*n* = 280)	All cases (*n* = 709)	
Age (median [IQR]) (yr)	71.3 (61.2–80.6)	75.2 (63.6–83.1)	78.0 (67.9–84.6)	75.2 (64.2–83.0)	<0.001
Male sex (no. [%])	125 (61.6)	135 (59.7)	203 (72.5)		0.005
Charlson score (median [IQR])	3 (1–5)	4 (2–6)	2 (0–3)	2 (1–5)	<0.001
Focus (no. [%])					<0.001
No focus/primary	119 (58.6)	90 (39.8)	116 (41.4)	325 (45.8)	
Skin/soft tissue	13 (6.4)	55 (24.3)	63 (22.5)	131 (18.5)	
Bone/joint	10 (4.9)	10 (4.4)	5 (1.8)	25 (3.5)	
Head and neck	0	1 (0.4)	0	1 (0.1)	
Lower respiratory tract	6 (3.0)	16 (7.1)	12 (4.3)	34 (4.8)	
Endovascular	3 (1.4)	1 (0.4)	1 (0.4)	5 (0.7)	
Central nervous system	0	0	1 (0.4)	1 (0.1)	
Intraabdominal	36 (17.7)	19 (8.4)	35 (12.5)	90 (12.7)	
Genitourinary	16 (7.9)	34 (15.0)	47 (16.8)	97 (13.7)	
Polymicrobial (no. [%])	72 (35.5)	76 (33.6)	91 (32.5)	239 (33.7)	0.8
Resistance to ≥1 class (no. [%])	27 (13.3)	20 (8.9)	20 (7.1)	67 (9.5)	0.08
Resistance (no./total no. tested [%])					
Ciprofloxacin	15/193 (7.8)	8/219 (3.7)	5/268 (1.9)	28/680 (4.1)	0.008
Gentamicin	16/200 (8.0)	7/224 (3.1)	6/279 (2.2)	29/703 (4.1)	0.007
Tobramycin	10/196 (5.1)	5/223 (2.2)	1/272 (0.4)	16/691 (2.3)	0.003
Meropenem	1/172 (0.6)	1/208 (0.5)	0/246	2/626 (0.3)	0.5
Trimethoprim-sulfamethoxazole	17/197 (8.6)	12 (5.4)	15 (5.4)	44/698 (6.3)	0.3

The most common foci of infection were skin and soft tissue (131 cases [18.5%]), genitourinary (97 cases [13.7%]), and intraabdominal (90 cases [12.7%]). Significant differences were observed for the foci of infection in relation to onset category (Table 1). Hospital-onset infections were most likely to be nonfocal and least likely to be of genitourinary or skin and soft tissue focus, whereas the greatest proportion of lower respiratory tract infections was among health care-associated cases, as shown in Table 1.

### Microbiology.

Overall, 239 episodes (33.7%) were associated with polymicrobial etiology, although this proportion did not vary significantly among onset categories (Table 1). There were no significant differences in sex, median age, Charlson score, or distribution of foci of infection among those with monomicrobial versus polymicrobial infections. Among the polymicrobial episodes, 239 had one coisolate, 88 had two, 30 had three, 9 had four, and 1 had five. Escherichia coli (74 cases [20.2%]), Enterococcus faecalis (52 cases [14.2%]), Proteus mirabilis (39 cases [10.6%]), Pseudomonas aeruginosa (29 cases [7.9%]), and Klebsiella pneumoniae (23 cases [6.3%]) were the most common species among the 367 coisolated organisms.

Resistance to one or more of the quinolones, co-trimoxazole, aminoglycosides, or carbapenems was observed in 67 cases (9.5%). A trend to increased prevalence of resistance was observed for hospital-onset cases (Table 1). However, the proportions of cases with resistance to one (32 cases [4.5%]), two (15 cases [2.1%]), or three (20 cases [2.8%]) classes did not differ according to onset (*P* = 0.127). Resistance to ceftriaxone, cefepime, ceftazidime, and piperacillin-tazobactam was found in 52/627 (8.3%), 10/556 (1.8%), 118/652 (18.1%), and 21/541 (3.9%) isolates tested, respectively.

### Outcomes.

Ninety-eight percent of cases (695 cases) were admitted to the hospital for management at the time of or within 2 days after BSI diagnosis. The median duration of the hospital stay was 11 days (6 to 28 days). A total of 150 patients died within 30 days after the Morganella morganii BSI, for an all-cause case fatality rate of 21.2%. A number of factors were found to be associated with adverse outcomes, as shown in Table 2. A logistic regression model (*n* = 705) was developed that had good fit (*P* = 0.2) and an area under the receiver operator characteristic curve of 0.7271. As shown in Table 3, older age, nonfocal infections, and comorbidities, including congestive heart failure, dementia, and cancer, were independently associated with increased risk of death.

**TABLE 2:** Factors associated with 30-day all-cause case fatality due to Morganella morganii BSIs

Factor	Data for patients who:			*P*
	Survived (*n* = 559)	Died (*n* = 150)	Overall (*n* = 709)	
Age (median [IQR]) (yr)	73.3 (62.6–82.0)	79.7 (71.2–86.5)	75.2 (64.2–83.0)	<0.0001
Male sex (no. [%])	365 (65.3)	98 (65.3)	463 (65.3)	0.5
Infection onset (no. [%])				0.6
Hospital	156 (27.9)	47 (31.3)	203 (28.6)	
Community associated	177 (31.7)	49 (32.7)	226 (31.9)	
Health care associated	226 (40.4)	54 (36.0)	280 (39.5)	
Charlson score (median [IQR])	2 (1–4)	4 (2–6)	2 (1–5)	<0.0001
Myocardial infarction (no. [%])	66 (11.8)	23 (15.3)	89 (12.6)	0.2
Congestive heart failure (no. [%])	118 (21.1)	49 (32.7)	167 (23.6)	0.005
Peripheral vascular disease (no. [%])	71 (12.7)	27 (18.0)	98 (13.8)	0.1
Cerebrovascular disease (no. [%])	46 (8.2)	17 (11.3)	63 (8.9)	0.2
Dementia (no. [%])	40 (7.2)	23 (15.3)	63 (8.9)	0.003
Chronic pulmonary disease (no. [%])	65 (11.6)	25 (16.7)	90 (12.7)	0.1
Rheumatic disease (no. [%])	15 (2.7)	5 (3.3)	20 (2.8)	0.4
Peptic ulcer disease (no. [%])	8 (1.4)	5 (3.3)	13 (1.8)	0.2
Liver disease (no. [%])	32 (5.7)	9 (6.0)	41 (5.8)	0.8
Diabetes mellitus (no. [%])	203 (36.3)	47 (31.3)	250 (35.3)	0.3
Plegia (no. [%])	43 (7.7)	8 (5.3)	51 (7.2)	0.4
Renal disease (no. [%])	155 (27.7)	53 (35.3)	208 (29.3)	0.09
Malignancy (no. [%])	91 (16.3)	45 (30.0)	136 (19.2)	<0.001
Metastatic cancer (no. [%])	31 (5.6)	23 (15.3)	54 (7.6)	<0.001
HIV positive (no. [%])	1 (0.2)	0	1 (0.1)	1
Focus of infection (no. [%])				0.016
No focus	229 (41.4)	89 (59.7)	318 (45.3)	
Soft tissue	109 (19.7)	22 (14.8)	131 (18.7)	
Bone and joint	22 (4.0)	3 (2.0)	25 (3.6)	
Head and neck	1 (0.2)	0	1 (0.1)	
Lower respiratory tract	28 (5.1)	6 (4.0)	34 (4.8)	
Endovascular	5 (0.9)	0	5 (0.7)	
Central nervous system	1 (0.2)	0	1 (0.1)	
Abdominal	73 (13.2)	17 (11.4)	90 (12.8)	
Urinary/pelvic	85 (15.4)	12 (8.1)	97 (3.8)	
Polymicrobial (no. [%])	185 (33.1)	54 (36.0)	239 (33.7)	0.5
Resistant (no. [%])	53 (9.5)	14 (9.3)	67 (9.5)	1

**TABLE 3:** Logistic regression modeling of factors associated with 30-day case fatality

Factor	Odds ratio (95% CI)	*P*
Age (per yr)	1.04 (1.01–1.05)	<0.001
Congestive heart failure	1.75 (1.14–2.69)	0.011
Dementia	1.98 (1.10–3.58)	0.023
No cancer	1	
Cancer without metastases	1.85 (1.06–3.25)	0.032
Cancer with metastases	4.02 (2.17–7.44)	<0.001
No focus of infection	2.28 (1.55–3.37)	<0.001

## DISCUSSION

This study identifies that Morganella morganii is a relatively infrequent but important cause of BSIs in our population. Older individuals, especially males and those with comorbidities, are at highest risk of developing Morganella morganii BSIs. One-fifth of patients who develop a Morganella morganii BSI will not survive past 30 days, and older patients, patients with nonfocal infections, and patients with a number of comorbidities are at highest risk of death. Although antimicrobial resistance did not increase during the study or increase the risk of death, the potential for this to add to the significant burden associated with these infections in future years merits ongoing surveillance.

The incidence rate of 9.2 cases per million population we observed is a novel observation. A previous population-based laboratory surveillance study investigating tribe *Proteeae* isolates in Calgary, Canada, found that Morganella morganii was associated with an overall incidence rate of 77 cases per million annually, with an incidence of bacteremia of approximately 3 cases per million per year (1). Other studies examining etiologies of BSIs in nonselected populations have not reported specific rates for Morganella morganii, to our knowledge (19, 20). It is also notable that the incidence we observed during surveillance increased over time. While there may be many explanations for this observation, it is likely that this reflects at least in part increased overall sampling in the population (21).

Previous hospital-based studies have reported on the clinical features and outcomes associated with Morganella morganii BSIs. Erlanger et al. most recently reported on 136 adults with Morganella morganii bacteremia who were admitted to a large university hospital in Israel in 1997 to 2014 (14). They found that 91 cases (68%) were acquired in a hospital or health care facility, that most patients were debilitated or highly comorbid, and that the 30-day case fatality rate was 52% (14). They further reviewed the literature and identified three other studies, including 109 cases from a medical center in Taiwan in 2003 to 2012 (16), 61 cases in a large tertiary hospital in South Korea in 1991 to 2000 (17), and 73 cases from another Taiwan medical center in 2002 and 2003 (18). Another study was also included in their review but was not limited to bacteremic cases (15). Those studies showed marked heterogeneity in results, with average ages ranging from 52 to 75 years, community-acquired infections ranging from 30% to 75%, and case fatality rates ranging from 15% to 42%. While these differences could in part reflect the definitions used and true differences in the populations under study, it is likely that selection bias arising from the conduct of these reviews at single hospitals played a significant role (22). Our study benefits from the inclusion of patients from all institutions, both small and large, and in urban and nonurban settings.

While our study benefits from a population-based design and includes a large cohort of patients surveyed over an extended period, there are some limitations. First, we did not have any data regarding the antimicrobial therapies prescribed; therefore, we could not examine this as a determinant of outcomes. However, we observed a relatively low rate of resistance and noted that its presence was not associated with survival outcomes. Second, we included only cases identified within the public system, such that patients managed in private institutions were not included in surveillance. Our results should thus be interpreted in this context, and the true rate of Morganella morganii BSIs is higher than we observed. Third, because this was a retrospective study, we were limited to the available data existing in our source databases. We did not have details regarding procedures to which patients might have been exposed, such as urinary tract instrumentation, which could be risk factors for both acquisition and adverse outcomes.

In conclusion, this novel study defines the epidemiology of Morganella morganii BSIs in a large population and identifies risk factors associated with adverse outcomes. While we observed that the incidence was increasing, antibiotic resistance was not. Given the current burden of disease and potential to increase, ongoing surveillance is warranted.

## MATERIALS AND METHODS

A retrospective, population-based, laboratory surveillance, cohort design was utilized. All residents of Queensland, Australia, who had BSIs due to Morganella morganii identified within the publicly funded health care system between 1 January 2000 and 31 December 2019 were included. This study was approved by the human research ethics committee at Royal Brisbane and Women’s Hospital, and a waiver of the requirement for individual consent was granted (LNR/2020/QRBW/62494).

All blood cultures for Morganella morganii during the surveillance period, including those obtained from community and institutional collection sites statewide, were first identified by Pathology Queensland. The BacT/Alert 3D system (bioMérieux, Durham, NC) was used throughout the study period with the exception that, as of 2018, the BacT/Alert VIRTUO system (bioMérieux) was used at the main central laboratory that manages culture submissions from the Greater Brisbane area and several rural Queensland sites. At Pathology Queensland, blood cultures are routinely incubated for 5 days before being discarded for no growth. BacT/Alert FA Plus (aerobic), FN Plus (anaerobic), and PF Plus (pediatric) medium bottles were used for culture. Species identification methods included the Vitek GN ID card, the API 20E test, and matrix-assisted laser desorption ionization–time of flight mass spectrometry (MALDI-TOF MS). Antibiotic susceptibility testing was performed using both an automated method (i.e., Vitek AST card) and the disc diffusion method according to recognized standards (CLSI or EUCAST) at the time of testing.

Once positive blood cultures were identified, linkages to statewide hospital admissions and death registries were performed to obtain clinical and outcome information. A series of algorithms and previously validated definitions were then applied to classify BSI episodes (23, 24). Incident episodes were defined by the first isolation of Morganella morganii per patient; repeat isolates within 30 days were deemed to represent the same incident episode. When one or more organisms were coisolated with Morganella morganii within a 48-h period, the BSI was classified as polymicrobial (25). Resistant organisms were defined as those nonsusceptible to one or more of ciprofloxacin/norfloxacin, co-trimoxazole, gentamicin/tobramycin, or imipenem/meropenem.

Admissions to any private or public institutions within the state were identified, and discharge diagnostic codes (ICD-10AM) were obtained. The index hospitalization included all encounters associated with the management of the BSI, including interhospital transfers within the state. The Registry of General Deaths as of 31 December 2020 was queried to confirm deaths in any setting within Queensland.

Hospital-onset BSIs were those for which the index blood culture was drawn more than 2 calendar days after admission or within 2 calendar days after hospital discharge (26). Community-onset BSIs were those for which the index culture was drawn in the community or within the first 2 days of hospital admission. Health care-associated BSIs were community-onset BSIs that occurred among nursing home residents or patients who had encounters at a health care institution within 30 days and/or admission to a hospital for more than 2 days within the 90 days prior to the index blood culture (27). Community-associated BSIs were community-onset BSIs that were not health care associated. Comorbid medical illnesses were defined using the Charlson Comorbidity Index (28, 29). A clinical focus was assigned based on review of diagnosis-related group and primary diagnosis hospital discharge codes.

Data were analyzed using Stata 17 (StataCorp, College Station, TX, USA). The primary unit of analysis was incident BSI episodes, and results were reported as age- and sex-standardized (to the 2019 Queensland population) annual rates per million population. Nonresidents of Queensland were excluded. Denominator data were stratified by age, sex, and hospital, and the health service area was obtained from Queensland Health using data available from the Australian Bureau of Statistics (30). The total annual number of sets of blood cultures performed by Pathology Queensland was obtained (21). IRRs with exact 95% CIs were calculated for group comparisons.

A multivariable logistic regression model was developed to examine factors associated with all-cause 30-day case fatality rates. Age, gender, onset classification, resistance, comorbidities, polymicrobial infection, and focus of infection were included in the initial model. Stepwise backward variable elimination was performed in order to develop the most parsimonious model. Calibration and discrimination were assessed using the Hosmer-Lemeshow test and the area under the receiver operator characteristic curve, respectively. *P* values of <0.05 were deemed to represent statistical significance for all comparisons.

### Data availability.

Data cannot be shared publicly due to institutional ethics, privacy, and confidentiality regulations. Data release for the purposes of research under Section 280 of the Public Health Act 2005 requires application to the Director-General (pha@health.qld.gov.au).
